# Frontostriatal regulation of brain circuits contributes to flexible decision making

**DOI:** 10.1038/s41386-025-02065-8

**Published:** 2025-02-14

**Authors:** Ying Duan, Zilu Ma, Pei-Jung Tsai, Hanbing Lu, Xiang Xiao, Danni Wang, Aslaan Siddiqi, Elliot A. Stein, Michael Michaelides, Yihong Yang

**Affiliations:** 1 Neuroimaging Research Branch, National Institute on Drug Abuse, Intramural Research Program, Baltimore, MD 21224 USA; 2 Department of Applied Psychology, Faculty of Arts and Sciences, Beijing Normal University at Zhuhai, Zhuhai, 519087 China

**Keywords:** Learning and memory, Decision

## Abstract

Deficits in behavioral or cognitive flexibility that are linked to altered activity in both cortical and subcortical brain regions, are often observed across multiple neuropsychiatric disorders. The medial prefrontal cortex (mPFC)-nucleus accumbens (NAc) pathway in rats plays a critical role in flexible control of behavior. However, the modulation of this pathway on activity and functional connectivity with the rest of the brain remains unclear. In this study, we first confirmed the role of the mPFC-NAc pathway in behavioral flexibility using a set-shifting task in rats and then evaluated the causal effects of mPFC-NAc activation induced by chemogenetic stimulation of the terminal axons of the NAc with DREADD expression on whole-brain activity and functional connectivity measured by functional MRI. mPFC-NAc activation improved performance on the set-shifting task by reducing perseverative errors. Additionally, stimulation of this pathway increased activity in a set of brain regions within the basal ganglia-thalamus-cortical loop network including NAc, thalamus, hypothalamus and various connected cortical regions, while also decreased functional connectivity strength of NAc-mPFC, NAc-secondary motor cortex (M2), and various cortical circuits. Moreover, performance on the set-shifting task was related to the functional connectivity strength of the above frontostriatal and cortical circuits. These findings provide insights into the link between specific frontostriatal circuits on decision making flexibility, which may inform potential future interventions for behavioral flexibility deficits.

## Introduction

Cognitive flexibility is defined as the ability to make appropriate behavioral adjustments in response to changing environmental demands [1]. Deficits in flexibility are often observed across several neurological diseases [2, 3], as well as psychiatric disorders [4–6]. As current treatments and interventions for these brain disorders have limited efficacy, deciphering the neural circuits underpinning flexibility and its dysregulation is crucial to uncover the neurobiological mechanisms shared by these disorders, which might lead to potential novel therapeutics.

As the executive function involving the ability to switch between multiple tasks or behavioral demands [7], flexibility has been measured by different behavioral tasks, most notably the Wisconsin Card Sorting Task (WCST) in humans [8, 9] and set-shifting tasks in preclinical models [10, 11]. A large body of studies using the WCST revealed impairments in cognitive flexibility in individuals with frontal cortex damage [12, 13] as well as stroke-induced subcortical damage in the basal ganglia and thalamus [14, 15]. Neuroimaging studies also revealed correlations between WCST performance with multiple networks, including the Executive Control Network, Default Mode Network and Salience Network [16–18].

While studies consistently show that large-scale brain networks underlie cognitive flexibility, development of therapeutic interventions requires a deeper understanding of how specific neural circuits influence brain networks to drive behavioral outcomes [19]. At a local circuit level, animal studies have shown that inactivation of the medial prefrontal cortex (mPFC) or its subcortical connection with the nucleus accumbens (NAc), disrupt flexibility in a set-shifting task [10, 20–22]. Recently, optogenetic excitation and inhibition of the mPFC-NAc pathway enhanced and disrupted flexibility in a cross-maze set-shifting task, respectively [23]. Here, we aim to identify the effects of mPFC-NAc pathway activation on whole-brain activity and functional connectivity to inform potential interventions for those with flexibility deficits.

Chemogenetic technology, Designer Receptors Exclusively Activated by Designer Drugs (DREADD), allows for direct activation of a particular region or circuit, subsequent measurement of “downstream” circuits, and its effect on behavior [24–28]. In this study, we combined excitatory hM3Dq DREADD activation and fMRI to evaluate the direct causal impact of mPFC-NAc activation on whole-brain activity and functional connectivity associated with a behavioral flexibility task. To specifically activate the mPFC-NAc pathway, we injected the DREADD into the mPFC and, as terminal axons with DREADD expression can be activated by local clozapine N-oxide (CNO) infusion, subsequently injected CNO into the NAc [29–31]. We assessed the effects of the mPFC-NAc pathway activation on a strategy set-shifting task conducted in an operant chamber, where rats initially learned to obtain rewards based on visual cues and were subsequently required to shift their strategy to spatial response-based discrimination [10]. In this task, animals were required to switch from the dimension they were currently engaged to another dimension with a new rule and then maintained it, which was an extradimensional set-shifting task to measure behavioral flexibility [11]. We subsequently used fMRI to evaluate the changes in whole-brain neural activity induced by mPFC-NAc pathway activation and the functional connectivity in NAc and mPFC related circuits. Finally, we examined the relationship between the fMRI measures and behavior performance.

## Materials and Methods

### Animals

A total 78 of adult male Sprague‐Dawley rats (300–360 g; Charles River) were used in the study. We only used male rats due to methodological considerations to fMRI procedure, as sex differences in brain anatomy and functional connectivity in rats [32] and ovarian hormone fluctuations in functional connectivity [33] could complicate experimental design under consistent conditions. Rats were housed two per cage before surgery and individually after surgery under a reverse 12:12 h light/dark cycle with ad libitum food and water. All procedures followed the NIH Guide for the Care and Use of Laboratory Animals (8th edition) and were approved by the NIDA Animal Care and Use Committee. Twelve rats were excluded in data analysis due to cannula misplacement (*n* = 2), loose head cap/damaged brain (*n* = 4), or failure to reach training criterion during the behavioral experiments (*n* = 6). Of the remaining 66 rats, 46 were used only for the behavioral experiment, while 20 rats yielded data for both behavioral and MRI experiments.

### Drugs

Clozapine-N-oxide (CNO; Tocris, Minneapolis, MN) was dissolved in 0.9% saline with 0.5% DMSO and bilaterally injected into the NAc at 1 mM, 0.5 µL/side [29, 30]. Vehicle (VEH) consisted of saline with 0.5% DMSO. Feraheme (15 mg/kg; AMAG Pharmaceuticals, Waltham, MA) was administered as contrast via tail vein injection.

### Experimental overview

**Fig. 1:**
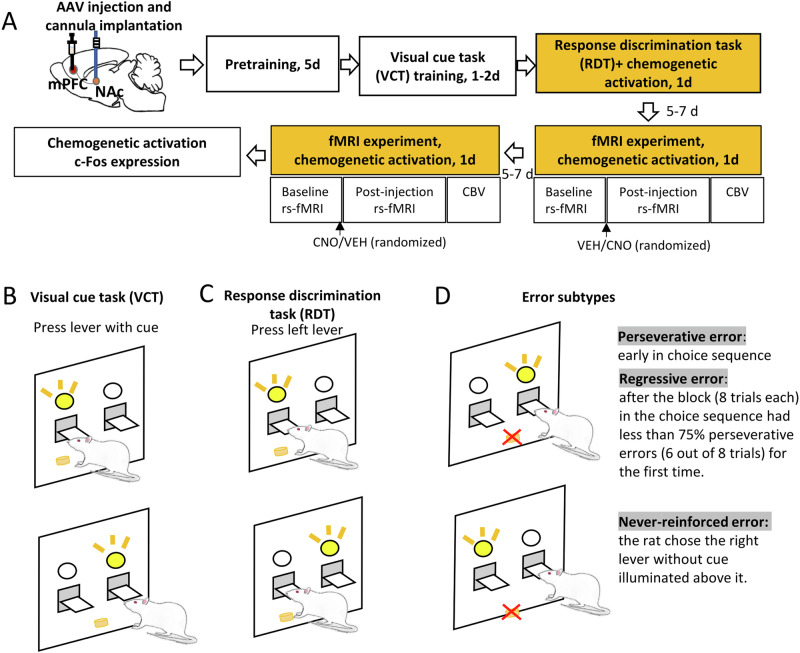
Experimental design and example of the strategy set-shifting task. **A** Experimental design. Rats were bilaterally injected with adeno-associated virus (AAV) expressing the excitatory DREADD hM3Dq or green fluorescent protein (GFP) as control (AAV5-syn-GFP) in the medial prefrontal cortex (mPFC) and bilateral cannulas were implanted 1 mm above the nucleus accumbens (NAc). After 2 weeks of recovery, rats underwent food pretraining for 5 days, visual-cue task (VCT) training for 1–2 days and response discrimination task (RDT) for 1 day. Rats were injected either with CNO or VEH into the NAc 20 min prior to the RDT. Five to seven days later, the fMRI experiment was conducted. In the scanner, rats underwent a baseline resting-state fMRI (rs-fMRI) scan to assess functional connectivity, followed by a CNO or VEH injection into the NAc. After 20 min, a post-injection rs-fMRI scan was performed followed by cerebral blood volume (CBV) to evaluate neural activity. The entire sequence was repeated 5–7 days later with reversed VEH or CNO injection order. Lastly, the rats were sacrificed for Immunofluorescence staining. **B** Example of VCT. The rat was required to respond on the lever that had a visual-cue stimulus light illuminated above it. **C** Example of RDT. The rat was required to cease the use of a visual-cue strategy, and instead use a spatial discrimination strategy to obtain food pellets. In this example, the rat was required to always press the left lever during the RDT. **D** Example of different subtypes of errors. The trials were separated into consecutive blocks of 8 trials each. *A perseverative error* was scored when the rat pressed the right lever when the light stimulus was on the right side in early stage of the choice sequence. However, if the proportion of such errors was less than 75% in a block (6 out of 8 trials), from that time point the error was scored as a *regressive error*. A *never-reinforced error* was scored when the rat chose the right lever without cue illuminated above it.

The experimental timeline is shown in Fig. 1A. Rats were bilaterally injected with adeno-associated virus (AAV) expressing the excitatory DREADD hM3Dq (AAV5-syn-hM3D-HA; Addgene, Watertown, MA) or green fluorescent protein (GFP) as control (AAV5-syn-GFP; Addgene) in the mPFC (AP +3.4 mm, ML ±0.7 mm, DV −3.8 mm) followed by cannulas placement 1 mm above the NAc (AP +1.5 mm, ML ±2.6 mm, DV −7 mm, angle 10°). After 2 weeks of recovery, rats underwent pretraining, Visual-Cue Task (VCT) and Response Discrimination Task (RDT), as well as MRI scanning. Detailed procedures are provided in the Supplementary Methods and Materials.

### Strategy acquisition and switching

Behavioral procedures were adapted from Floresco et al. [10]. To maintain motivation without induing potential stress from food restriction [34], rats had free food access and received palatable food pellets (45 mg, Ain-76A, TestDiet, Richmond, IN) as reward during training.

#### Pretraining

Rats underwent a fixed ratio 1 (FR1) schedule for 5 days, with two 90-trial sessions per day. A single lever (side counterbalanced) was presented per session. Every trial lasted 20 s beginning with houselight illumination. Responses within 10 s delivered a pellet.

#### VCT

Both levers were presented, and rats had to press the lever with an illuminated cue light within 10 s to receive a pellet. Sessions continued until rats achieved 8 consecutive correct responses or completed 200 trials, with an additional session allowed. Rats failing to meet the criterion within two sessions were excluded.

#### RDT

Rats had to press the lever opposite their predetermined side bias regardless of the cue light illumination. Sessions continued until rats achieved 10 consecutive correct responses or complete 200 trials.

### MRI experiments

Following established protocols [35–37], anesthesia was induced with 2.5% isoflurane and dexmedetomidine (0.015 mg/kg, subcutaneous). During imaging, rats was anesthetized with isoflurane (0.75–0.5%) and dexmedetomidine (0.015 mg/kg/h, subcutaneous). Structural imaging used a T2-weighted rapid acquisition with relaxation enhancement sequence. Functional imaging used single-shot gradient-echo EPI. The baseline scan was collected followed by VEH or CNO injection, after which three 10-min post-injection scans were performed following a 20-min delay. For CBV imaging, pre- and post-contrast scans were acquired using a multi-gradient echo (MGE) sequence.

### Statistical analyses

#### Behavioral data analyses

Pretraining data were analyzed using repeated measures ANOVA for comparisons across sessions and between groups. VCT training data were analyzed using unpaired t-test for comparisons between groups. RDT data were analyzed using two-way ANOVA with GROUP (control, hM3Dq) as a between-subject factor and TREATMENT (VEH, CNO) as within-subject factors followed by Bonferroni post hoc test. Statistical significance was preset at *p* < 0.05.

#### CBV analyses

Data were processed using an in-house MATLAB based processing pipeline [37, 38]. Specifically, MGE data was fitted to a single-exponential delay model: $S={S}_{0}\times {e}^{(-{TE}\times {R}_{2}^{* })}$. Here *S*_0_ represents MRI signal at *TE* = 0; *R*_2_* represents the transverse-relaxation-rate. Since Feraheme is an intravascular contrast agent, change in the *R*_2_* pre- and post-MION injection (Δ*R*_2_*) is proportional to CBV [39]. Voxel-wise *t*-test was used to compare difference in CBV levels between CNO and VEH injection in the hM3Dq or control groups separately. Results were corrected for multiple comparisons using Monte Carlos simulation in AFNI (corrected *p* < 0.05, uncorrected *p* < 0.05 and cluster size >40).

#### Functional connectivity analyses

Data were preprocessed using a conventional pipeline [35–37, 40] including distortion correction, skull stripping, motion correction, co-registration to a template, noise component removal, band-pass filtering (0.01–0.1 Hz), and spatial smoothing (FWHM = 0.8 mm). Seed-based functional connectivity maps were submitted to a linear mixed-effects model ANOVA, with GROUP (control, hM3Dq) as a between-subject factor, SESSION (baseline, post-injection) and TREATMENT (VEH, CNO) as within-subject factors. Results were corrected for whole-brain multiple comparisons (corrected *p* < 0.05, uncorrected *p* < 0.05 and cluster size >27 voxels based on Monte Carlo simulations in AFNI). Modified atlas images [41] are used for MRI image superimposition and region identification. Principal component analysis (PCA) followed by K-mean clustering on functional connectivity of the brain circuits with significant brain voxels was performed to determine different networks using Prism 10 and R 4.4.1. Lastly, correlations between average functional connectivity in each network and set-shifting behavior (number of trials to reach criterion) were calculated. We selected this behavioral parameter as it reflected the general performance during the set-shifting procedure. Statistical significance was corrected for multiple comparisons using the Bonferroni method (*p* < 0.025 for functional connectivity seed with NAc or M2, *p* < 0.017 for functional connectivity seed with mPFC).

## Results

### Activation of mPFC-NAc projection promotes set-shifting behavior and increases neural activity in the basal ganglia-thalamus-cortical loop

**Fig. 2:**
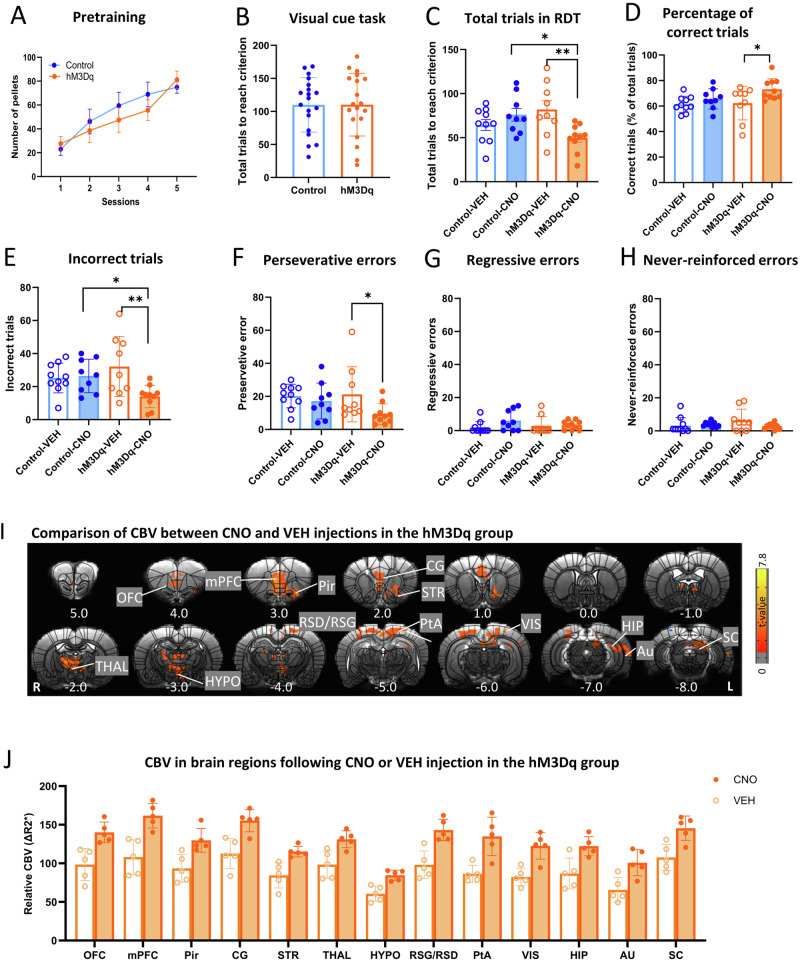
Activation of mPFC-NAc pathway promoted visual cue task (VCT) shifting to response discrimination task (RDT). **A** There was no significant difference in the pretraining phase between the hM3Dq (*n* = 19) and control (*n* = 19) groups. **B** There was no significant difference in number of total trails to reach criterion between the two groups in VCT training. **C** Activation of the mPFC-NAc pathway significantly reduced the number of total trials needed to reach the criterion in RDT (*n* = 9–10 per group). Data were analyzed by two-way ANOVA followed by Bonferroni post hoc test. *p < 0.05, hM3Dq-CNO vs. control-CNO; **p < 0.01, hM3Dq-CNO vs. hM3Dq-VEH. **D** Activation of the mPFC-NAc pathway significantly increased the percentage of correct trials in RDT (*n* = 9–10 per group). Data were analyzed by two-way ANOVA followed by Bonferroni post hoc test. *p < 0.05, hM3Dq-CNO vs. hM3Dq-VEH. **E** Activation of the mPFC-NAc pathway significantly reduced the number of incorrect trials in RDT (*n* = 9–10 per group). Data were analyzed by two-way ANOVA followed by Bonferroni post hoc test. *p < 0.05, hM3Dq-CNO vs. control-CNO; **p < 0.01, hM3Dq-CNO vs. hM3Dq-VEH. **F** Activation of the mPFC-NAc pathway significantly decreased perseverative errors in the hM3Dq group but not the control group. Two-way ANOVA followed by Bonferroni post hoc test. *p < 0.05, hM3Dq-CNO vs. hM3Dq-VEH. **G** Activation of the mPFC-NAc pathway had no effects on regressive errors. **H** Activation of the mPFC-NAc pathway had no effects on never-reinforced errors. **I** The comparison of cerebral blood volume (CBV) maps between VEH and CNO injections in the hM3Dq group (*n* = 5). Data were analyzed by voxel-wise *t*-test. Results were corrected for whole-brain multiple comparisons at *p*_corr_ < 0.05. **J** CNO injection in the NAc increased CBV in the orbitofrontal cortex (OFC), mPFC, piriform cortex (Pir), cingulate cortex (CG), striatum (STR), thalamus (THAL), hypothalamus (HYPO), retrosplenial dysgranular/granular cortex (RSD/RSG), parietal association cortex (PtA), visual cortex (VIS), hippocampus (HIP), auditory cortex (AU) and superior colliculus (SC). Coordinates represent distance relative to bregma (in millimeters). Error bars in the figures represent the SEM. VEH vehicle, CNO clozapine N-oxide.

We first examined differences in behaviors between excitatory DREADD (hM3Dq) and control groups in different phases of learning. During pretraining (Fig. 2A), Two-way ANOVA showed a significant SESSION effect (*F*_4,144_ = 19.19, *p* < 0.001) but no GROUP effect (*F*_1,36_ = 0.23, *p* = 0.64) or GROUP × SESSION interaction (*F*_4,144_ = 1.042, *p* = 0.39). In addition, there was no significant difference in number of total trials to reach criterion between the two groups during VCT training (*t*-test: *t* = 0.022, *p* = 0.98) (Fig. 2B), suggesting that there was no difference in learning abilities between the hM3Dq and control groups.

On the day of switching to the RDT, either CNO or VEH was microinjected into the NAc 20 min before testing. Two-way ANOVA revealed a significant GROUP (hM3Dq, control) × TREATMENT (VEH, CNO) interaction effect on the number of total trials required to reach criterion (*F*_1,34_ = 9.31, *p* < 0.05). Post hoc analysis using Bonferroni test demonstrated that mPFC-NAc activation (hM3Dq-CNO) significantly decreased total trials compared with the control-CNO group (*p* < 0.05) and hM3Dq-VEH group (*p* < 0.05) (Fig. 2C). The above result indicates that mPFC-NAc activation facilitates strategy switching from VCT to RDT. For the percentage of correct trials, significant treatment effect was found (Two-way ANOVA, TREATMENT effect, *F*_1,34_ = 6.34, *p* < 0.05) and post hoc test showed mPFC-NAc activation increased percentage of correct trials compared with the hM3Dq-VEH group (*p* < 0.05) (Fig. 2D). Significant GROUP × TREATMENT interaction effect was also found in number of incorrect trials (*F*_1, 34_ = 6.57, *p* < 0.05; Bonferroni post hoc test: hM3Dq-CNO vs. hM3Dq-VEH, *p* < 0.01; hM3Dq-CNO vs. control-CNO, *p* < 0.05) (Fig. 2E). For the subtypes of errors, mPFC-NAc activation decreased number of perseverative errors (Two-way ANOVA: TREATMENT effect, *F*_1, 34_ = 4.55, *p* < 0.05; Bonferroni post hoc test: hM3Dq-CNO vs. hM3Dq-VEH, *p* < 0.05) (Fig. 2F) but neither regressive errors nor never-reinforced errors (Fig. 2G, H). Using a separate cohort of animals, we examined the effect of mPFC-NAc activation on the initial discrimination learning. The CNO or VEH was injected 20 min before VCT (Fig. S1A). The results showed that DREADD activation of mPFC-NAc did not affect initial visual cue learning (Fig. S1B–D), suggesting that mPFC-NAc was involved in in set-shifting specifically but not initial discrimination learning.

To verify methodological integrity, hM3Dq expression was detected in both mPFC and NAc (Fig. S2A), suggesting the presence of a direct projection from mPFC to the NAc. Compared with the VEH group (rats expressing either hM3Dq or control GFP), CNO microinjection significantly increased c-Fos expression in the hM3Dq group but not the control group (One way ANOVA, *F*_2,16_ = 6.73, *p* < 0.01; Tukey test: *p* < 0.01, hM3Dq-CNO vs. VEH group; *p* = 0.43, control-CNO vs. VEH group), suggesting specific chemogenetic activation of the mPFC-NAc pathway (Fig. S2B). Figure S2C shows canula placements for all rats including in the behavioral experiment.

In addition, CBV was detected to map neural activity following DREADD activation. In the group with mPFC hM3Dq expression, CNO injected into the NAc significantly increased activity (voxel-wise *t*-test, corrected *p* < 0.05) in the striatum (STR) as well as several cortical regions, including orbitofrontal cortex (OFC), mPFC, piriform cortex (Pir), cingulate cortex (CG), retrosplenial dysgranular/granular cortex (RSD/RSG), parietal association cortex (PtA), visual cortex (VIS) and auditory cortex (AU). Elevated activity was also found in the thalamus (THAL), hypothalamus (Hypo), hippocampus (HIP) and superior colliculus (SC), compared with the VEH injection (Fig. 2I). CBV values from clusters of significant voxels were extracted (Fig. 2J). In contrast, there no significant difference between CNO and VEH in the control.

Together, these results demonstrated that parallel with the changes in number of total trials and percentage of correct trails, activation of mPFC-NAc pathway mainly decreased the number of perseverative errors. Additionally, mPFC-NAc increased neural activity in a set of brain regions in the basal ganglia-thalamus-cortical loop.

### Activation of mPFC-NAc pathway decreased NAc functional connectivity with mPFC and M2

**Fig. 3:**
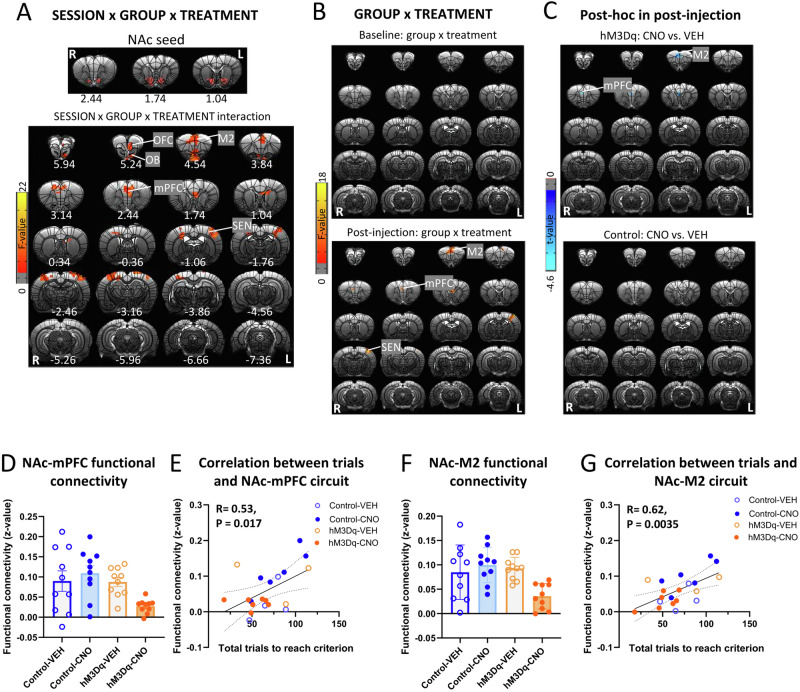
Activation of mPFC-NAc pathway decreased functional connectivity in NAc circuits. **A** The top shows the location of NAc seed, which was defined according to the cannula tip in each rat. SESSION (baseline, post-injection) × GROUP (hM3Dq, control) × TREATMENT (CNO, VEH) ANOVA showed a significant interaction effect in functional connectivity of NAc with orbitofrontal cortex (OFC), olfactory bulb (OB), secondary motor cortex (M2), mPFC and sensory cortex (SEN). Results were corrected for whole-brain multiple comparisons at *p*_corr_ < 0.05. **B** GROUP × TREATMENT ANOVA showed a significant interaction effect in functional connectivity of NAc with M2, mPFC and SEN after injection. However, there was no significant effect in the baseline. Results were corrected for whole-brain multiple comparisons at *p*_corr_ < 0.05. **C** Post hoc analyses found a significant difference in functional connectivity of NAc with mPFC and M2 between rats with CNO or VEH injection in the hM3Dq group but not the control group. Results were corrected for whole-brain multiple comparisons at *p*_corr_ < 0.05. **D** CNO injection decreased functional connectivity of NAc-mPFC in the hM3Dq group but not the control group. **E** Functional connectivity of NAc-mPFC was positively correlated with number of total trials to reach the criterion. Statistical significance was preset at *p*_uncorr_ < 0.025 for multiple comparisons correction. **F** CNO injection decreased functional connectivity of NAc-M2 in the hM3Dq group but not the control group. **G** Functional connectivity of NAc-M2 was positively correlated with number of total trials to reach the criterion. Statistical significance was preset at *p*_uncorr_ < 0.025 for multiple comparisons correction. *N* = 10 per group. Error bars in the figures represent the SEM. Coordinates represent distance relative to bregma (in millimeters). VEH vehicle, CNO clozapine N-oxide.

We next examined functional connectivity changes induced by mPFC-NAc pathway activation. To obtain functional connectivity map with the NAc seed, we defined individual NAc seed with eight voxels (0.375 × 0.375 × 0.7 mm^3^ per voxel) below the bilateral cannula tip in each rat (Fig. 3A). GROUP (hM3Dq, control) × SESSION (baseline, post-injection) × TREATMENT (CNO, VEH) ANOVA showed a significant 3-way interaction in functional connectivity of NAc with mPFC and other brain regions including secondary motor cortex (M2), olfactory bulb (OB), OFC and sensory cortex (SEN) (Fig. 3A). To address interaction effect driven by session, we performed follow-up GROUP × TREATMENT ANOVAs in different sessions separately. Figure 3B showed a significant two-way interaction in functional connectivity of NAc with M2, mPFC and SEN in the post-injection, but not pre-injection scan. To further compare CNO and VEH injection in different groups, follow-up *t*-tests showed that CNO injection decreased NAc-M2 and NAc-mPFC functional connectivity strength in the hM3Dq group but not the control group (Fig. 3C). Table S1 showed mean and standard deviation (SD) values for functional connectivity of significant clusters and corresponding test statistics. Figure 3D, F illustrated the decreased functional connectivity of the NAc-mPFC and NAc-M2 circuits, respectively, following CNO-injections in the hM3Dq group.

Finally, we correlated the functional connectivity strength of the NAc-mPFC and NAc-M2 circuits with number of total trials to reach criterion. The functional connectivity of both the NAc-mPFC and NAc-M2 circuits was positively correlated with number of total trials to reach criterion (Fig. 3E, G), suggesting that the strength of connections between these circuits was, at least in part, responsible for set-shifting behaviors.

### Activation of mPFC-NAc pathway decreased functional connectivity strength in mPFC-related circuits

**Fig. 4:**
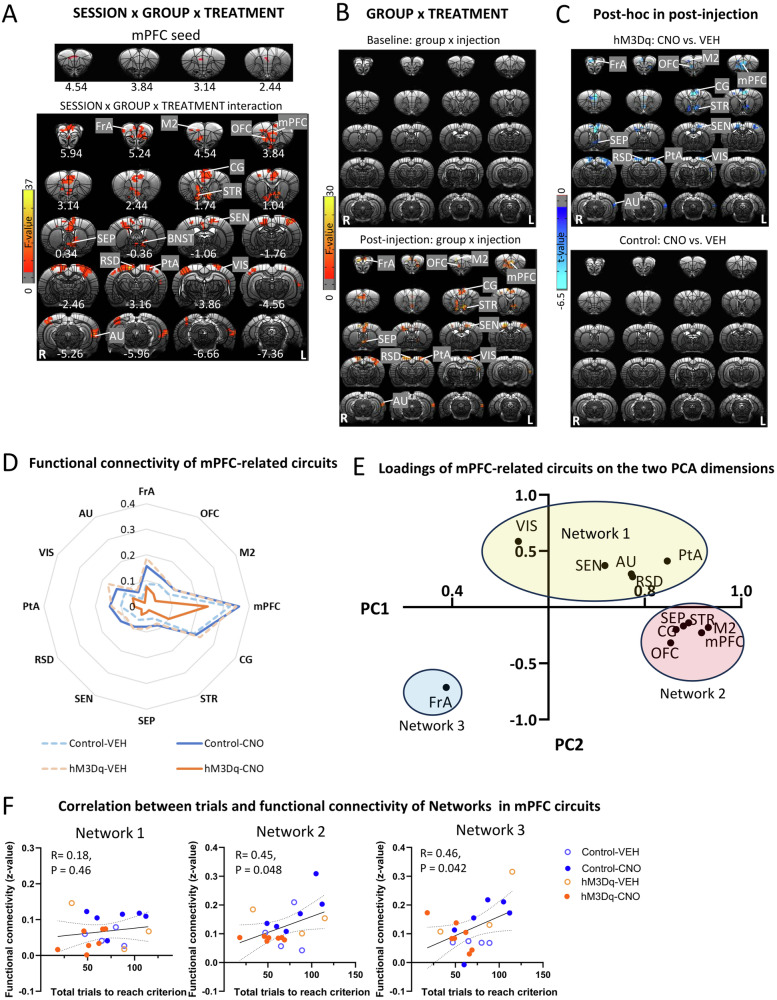
Activation of mPFC-NAc pathway decreased functional connectivity in mPFC circuits. **A** The top showed mPFC seed, which was defined as the region showing significance in the analysis on functional connectivity with the NAc seed. SESSION (baseline, post-injection) × GROUP (hM3Dq, control) × TREATMENT (CNO, VEH) ANOVA showed a significant interaction effect in functional connectivity of mPFC with frontal association cortex (FrA), secondary motor cortex (M2), orbitofrontal cortex (OFC), mPFC, cingulate cortex (CG), striatum (STR), septum (SEP), sensory cortex (SEN), bed nucleus of the stria terminalis(BNST), retrosplenial dysgranular cortex (RSD), parietal association cortex (PtA), visual cortex (VIS) and auditory cortex (AU). Results were corrected for whole-brain multiple comparisons at *p*_corr_ < 0.05. **B** GROUP × TREATMENT ANOVA showed a significant interaction effect in functional connectivity of mPFC with FrA, OFC, M2, mPFC, CG, STR, SEP, SEN, RSD, PtA, VIS and AU after injection. However, there was no significant effect in the baseline. Results were corrected for whole-brain multiple comparisons at *p*_corr_ < 0.05. **C** Post hoc analyses showed significant differences in functional connectivity of mPFC with FrA, OFC, M2, CG, STR, SEP, SEN, RSD, PtA, VIS and AU between rats with CNO or VEH injection in the hM3Dq group but not the control group. Results were corrected for whole-brain multiple comparisons at *p*_corr_ < 0.05. **D** Chemogenetic activation of mPFC-NAc pathway decreased functional connectivity of the above mPFC-related circuits. **E** Loadings of mPFC-related circuits on the two PCA dimensions were clustered into three networks. **F** Functional connectivity of the Network 1, 2 and 3 was not related to number of total trials to reach criterion. Statistical significance was preset at *p*_uncorr_ < 0.017 for multiple comparisons correction. *N* = 10 per group. Coordinates represent distance relative to bregma (in millimeters). VEH vehicle, CNO clozapine N-oxide.

A whole-brain voxel-wise functional connectivity ANOVA using the significant mPFC area as a seed (Fig. 3C), revealed a significant GROUP (hM3Dq, control) × SESSION (baseline, post-injection) × TREATMENT (CNO, VEH) interaction of mPFC with several cortical and striatal regions (Fig. 4A). Follow-up GROUP × TREATMENT ANOVAs indicated that the above circuits only showed a significant interaction in the post-injection but not baseline session (Fig. 4B), such that activation of mPFC-NAc pathway decreased the functional connectivity of mPFC with frontal association cortex (FrA), OFC, M2, mPFC, CG, VMPFC, STR, septum (SEP), SEN, RSD, PtA, VIS, and AU (Fig. 4C). Table S2 showed mean and SD values for functional connectivity of significant clusters and corresponding test statistics. Figure 4D illustrated the decreased functional connectivity of the above circuits following CNO-injections in the hM3Dq group.

PCA of these functional connectivity data revealed two principal components, which explained 76% of the variance, and loadings of mPFC-related circuits on the two PCA dimensions could be clustered into three “networks” (Fig. 4E). Network 1 included VIS, SEN, AU, RSG and PtA; Network 2 included STR, SEP, CG, mPFC, M2, and OFC; and Network 3 only included FrA. However, there were no significant correlations between functional connectivity of networks with the set-shifting behavior (Fig. 4F).

### Activation of mPFC-NAc pathway decreased functional connectivity strength in M2-related circuits

**Fig. 5:**
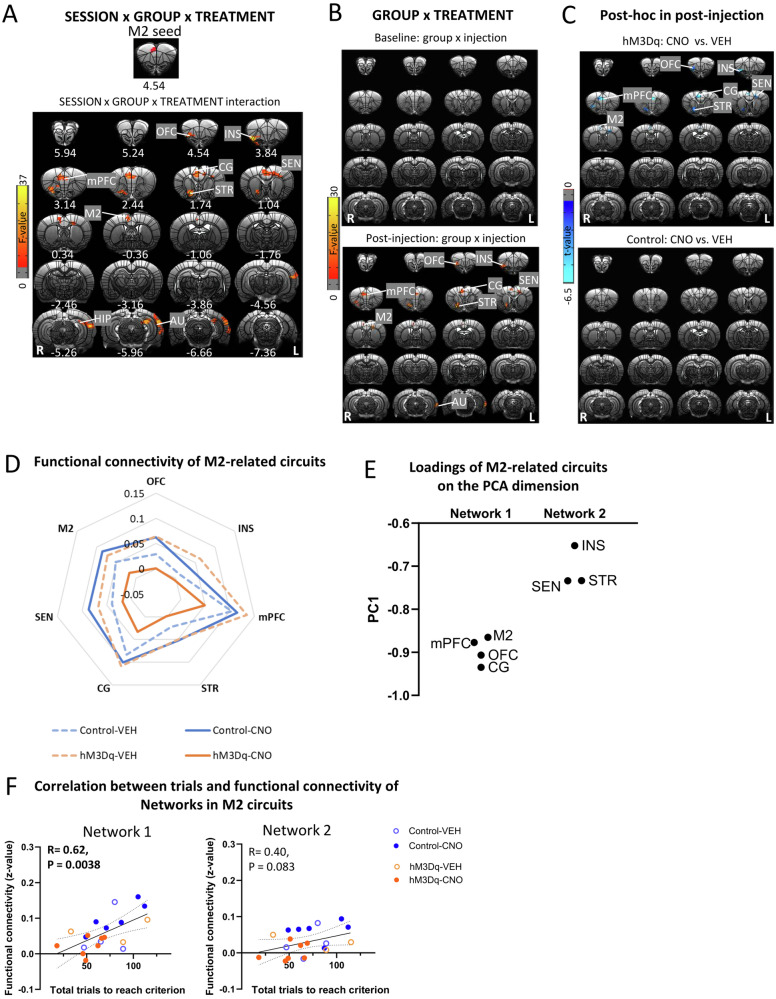
Activation of mPFC-NAc pathway decreased functional connectivity of M2 circuits. **A** The top showed M2 seed, which was defined as the region showing significance in the analysis on functional connectivity with the NAc seed. SESSION (baseline, post-injection) × GROUP (hM3Dq, control) × TREATMENT (CNO, VEH) ANOVA showed a significant interaction effect in functional connectivity of M2 with orbitofrontal cortex (OFC), insula (INS), cingulate cortex (CG), striatum (STR), sensory cortex (SEN), M2, hippocampus (HIP) and auditory cortex (AU). Results were corrected for whole-brain multiple comparisons at *p*_corr_ < 0.05. **B** GROUP × TREATMENT ANOVA showed a significant interaction effect on functional connectivity of M2 with OFC, INS, mPFC, CG, STR, SEN and M2 after injection. However, there was no significant effect in the baseline. Results were corrected for whole-brain multiple comparisons at *p*_corr_ < 0.05. **C** Post hoc analyses showed significant differences in functional connectivity of M2 with OFC, INS, mPFC, CG, STR, SEN and M2 between rats with CNO or VEH injection in the hM3Dq group but not the control group. Results were corrected for whole-brain multiple comparisons at *p*_corr_ < 0.05. **D** Chemogenetic activation of mPFC-NAc pathway decreased functional connectivity of the above M2-related circuits. **E** Loadings of M2-related circuits on the PCA dimension were clustered into two networks. **F** Functional connectivity of Network 1 but not Network 2 significantly correlated with number of total trials to reach criterion. Statistical significance was preset at *p*_uncorr_ < 0.025 for multiple comparisons correction. *N* = 10 per group. Coordinates represent distance relative to bregma (in millimeters). VEH vehicle, CNO clozapine N-oxide.

To examine potential changes in M2-related circuits induced by activating mPFC-NAc pathway, we conducted a whole-brain, voxel-wise functional connectivity analysis using the significant M2 area in Fig. 3C as a seed. GROUP (hM3Dq, control) × SESSION (baseline, post-injection) × TREATMENT (CNO, VEH) ANOVA showed a significant interaction in functional connectivity of M2 with several cortical and striatal regions including OFC, insula (INS), mPFC, CG, M2, STR, hippocampus (HIP) and AU (Fig. 5A). Follow-up GROUP × TREATMENT ANOVAs indicated that the M2 based circuits with OFC, INS, mPFC, M2, CG and STR all showed significant interaction in the post-injection but not baseline session (Fig. 5B). Follow-up *t*-tests revealed that for all the above circuits, except M2-AU, there was a significant difference between CNO and VEH injection in the hM3Dq group but not the control group (Fig. 5C). Table S3 showed mean and SD values for functional connectivity of significant clusters and corresponding test statistics. Figure 5D illustrated the decreased functional connectivity of the above circuits following CNO-injections in the hM3Dq group.

PCA of these functional connectivity data revealed one principal component, and loadings of M2-related circuits on the dimension could be clustered into two “networks”, with those connecting with mPFC, OFC, CG and M2 clustered into Network 1 and circuits with INS, SEN and STR clustered into Network 2 (Fig. 5E). Interestingly, functional connectivity in Network 1 but not in Networks 2, significantly correlated with the number of trials to reach criterion and the number of total error trials (Fig. 5F, G), suggesting that these circuits in Network 1 may acted together in a network to help regulate set-shifting behavior.

## Discussion

Deficits in cognitive flexibility are observed in multiple neuropsychiatric disorders [1, 2, 4–6, 42]. The mPFC-NAc pathway has been demonstrated to play an important role in behavioral flexibility in preclinical models [10, 20, 21, 23]. However, how this pathway interacts with other brain networks to modify behavior remains poorly understood. In the current study, using chemogenetic tools with a behavioral and fMRI readouts we show that mPFC-NAc activation increased neuronal activity in a broad distribution of brain regions including the NAc, thalamus, hypothalamus and various cortical regions, while also decreased the functional connectivity between the NAc and mPFC and M2, in addition to “downstream” mPFC and M2-related circuits. Critically, performance on a set-shifting task was related to the functional connectivity strength of these frontostriatal and cortical-cortical circuits.

### MPFC-NAc pathway is involved in set-shifting updating via suppressing old rules

The learning processes during set-shifting task can be inferred by analyzing different types of errors: suppressing old modes of responding (perseverative errors), exploring novel strategies (never-reinforced errors) and maintaining novel strategies once perseveration has ceased (regressive errors) [20]. Our results demonstrate that mPFC-NAc pathway activation promotes set-shifting by selectively decreasing perseverative errors, suggesting the improvement in flexible behavior is due to suppressing old decision models. This result is in line with a mPFC-NAc disconnection study in which unilateral bupivacaine infused into the PFC combined with a contralateral infusion of baclofen + muscimol into the NAc impaired flexibility by increasing perseverative errors [21]. However, using optogenetic tools to activate mPFC-NAc pathway, Cui et al. found that both perseverative and regressive errors were significantly altered in the manipulated groups [23]. This inconsistency may be explained by methodological and/or species distinctions in that Cui et al. used unilateral optogenetic manipulations in mice [23], while we activated mPFC-NAc pathway bilaterally using DREADD in rats, which may have led to different downstream circuits or interactions between hemispheres. Nevertheless, our behavioral results support the critical role of mPFC-NAc pathway in flexibility, mainly by suppressing old cognitive models.

It is worth noting that we did not examine the effect of mPFC-NAc activation on strategy from RDT to VCT. A previous study reported inconsistencies between VCT-to-RDT and RDT-to-VCT in the operant chamber likely due to the difference in task difficulty between initial learning strategies [10]. Future studies should adjust pretraining and initial response discrimination protocols to ensure comparable difficulty levels across strategies.

### Decreased functional connectivity of frontostriatal circuits is associated with improved flexibility

We found that activation of the mPFC-NAc pathway decreased functional connectivity of NAc with mPFC and M2 and related cortical circuits. This observation is in line with a study showing that chemogenetic activation of somatosensory cortex in mice decreased functional connectivity between somatosensory cortex and its monosynaptically connected cortical areas [43]. As both mPFC and M2 have direct projections with NAc [44], activating mPFC-NAc pathway may disrupt synchrony between NAc and cortical regions, leading to reduced functional connectivity synchrony despite higher local neural activity in these regions.

Moreover, our results showed that higher NAc-mPFC functional connectivity was associated with worse flexibility performance. The rodent mPFC is thought functionally analogous to the primate lateral prefrontal cortex [45], and our result is consistent with a clinical study showing that the primary role of the lateral prefrontal cortex is associated with performance on WCST [46]. Notably, we also found that the NAc-M2 circuit was related to behavioral flexibility. The M2 in rodents is a homolog of the premotor cortex in primates [47], and its location adjacent to medial prefrontal cortex and primary motor cortex suggests that it may function as a cognitive-motor interface [47, 48]. Recently, bilateral inactivation of M2 selectively impaired the shift into sound-guided actions [49], supporting the role of M2 in flexible behavior responding. However, the regulatory circuit mechanism of M2, such as the M2-NAc pathway, involved in flexibility still needs to be tested.

### Cortical networks contribute distinctly to behavioral flexibility

We also assessed the effects of mPFC-NAc pathway activation on cortical circuits associated with flexibility. Using PCA analyses of mPFC-related circuits and M2 related circuits, only Network 1 in M2-circuits comprised cortical-cortical circuits showed significant correlation with set-shifting behavior. It likely reflects the role of M2 in interacting with other cortical regions to participate in high-order execution functions since all of the cortical regions are reciprocally connected with M2 [50]. Additionally, chemogenetic attenuation of OFC projections into M2 biased novel lever exploration, suggesting that OFC-M2 pathway participated in new rule learning [51]. However, as previously mentioned, the circuit mechanisms of M2, such as M2-mPFC pathway, in a set-shifting task warrants further investigation.

Interestingly, functional connectivity of the networks including several sensory areas in both mPFC and M2 related circuits, was not associated with behavioral flexibility. This appears to be inconsistent with a classic hypothesis that mPFC plays a role in set-shifting tasks by mediating top-down control of sensorimotor processing [50, 52]. However, a recent study indicated that activity of mPFC neurons encoding the response in a set-shifting task was detected only after trial completion but not during the presence of whisker and odor stimuli, suggesting the critical role of mPFC in encoding feedback information but not modulating sensorimotor response [51].

### Method considerations and limitations

Activation of the mPFC-NAc pathway increased neural activity in a basal ganglia-thalamic-cortical network, including medial parts of thalamus, hypothalamus and several cortical areas including the mPFC, orbitofrontal cortex, infralimbic, cingulate, visual and auditory cortex. Consistent with our findings, another study demonstrated that chemogenetic activation of striatal neurons in mice altered neural activity of downstream regions including medial thalamus and cortical regions [25]. However, it is noteworthy that stimulation of NAc terminals can induce antidromic activity to increase neuronal activity in the mPFC [53], raising the question of whether the changes in cortical activity are directly due to NAc activation or mediated indirectly through a basal ganglia-thalamus-cortex pathway.

In contrast, other studies have shown no significant differences in functional connectivity after DREADD manipulation in striatal neurons or projections from ventral tegmental area to NAc or mPFC [24, 25]. Besides the differences in regions/pathways of manipulation, important methodological differences may help account for their null result, including their use of 1% isoflurane. Evidence shows that functional connectivity decreases with higher isoflurane levels [54], while our previous studies, which used 0.5% of isoflurane combined with a low dose of dexmedetomidine during imaging acquirement have successfully identified the classic Default Mode and Salience Networks in rats [35, 55], suggesting that this anesthesia regimen allowed us to observe more robust changes in functional connectivity induced by DREADD activation. However, it should be generally cautious when interpreting the relationship between fMRI data and behavioral outcomes under anesthesia. Future studies employing awake fMRI protocols could help validate and extend the current findings.

## Conclusion

The current study demonstrated that activation of the mPFC-NAc pathway enhanced behavioral flexibility by reducing perseverative errors and decreased functional connectivity in frontostriatal and other cortical circuits. These findings provide insights into the causal effects of frontostriatal pathway activation to behavioral flexibility, which may inform potential future interventions for those with behavioral flexibility deficits.

## Supplementary information

Supplementary methods and materials

Supplementary Tables S1, S2, S3

## Data Availability

The behavioral and imaging data are available upon request (YY).

## References

[1] Uddin LQ. Cognitive and behavioural flexibility: neural mechanisms and clinical considerations. Nat Rev Neurosci. 2021;22:167–79. 33536614 10.1038/s41583-021-00428-w PMC7856857

[2] Lange F, Bruckner C, Knebel A, Seer C, Kopp B. Executive dysfunction in Parkinson’s disease: a meta-analysis on the Wisconsin Card Sorting Test literature. Neurosci Biobehav Rev. 2018;93:38–56. 29944959 10.1016/j.neubiorev.2018.06.014

[3] Guarino A, Favieri F, Boncompagni I, Agostini F, Cantone M, Casagrande M. Executive functions in Alzheimer disease: a systematic review. Front Aging Neurosci. 2018;10:437. 30697157 10.3389/fnagi.2018.00437 PMC6341024

[4] D’Cruz AM, Ragozzino ME, Mosconi MW, Shrestha S, Cook EH, Sweeney JA. Reduced behavioral flexibility in autism spectrum disorders. Neuropsychology. 2013;27:152–60. 23527643 10.1037/a0031721 PMC3740947

[5] Waltz JA, Kasanova Z, Ross TJ, Salmeron BJ, McMahon RP, Gold JM, et al. The roles of reward, default, and executive control networks in set-shifting impairments in schizophrenia. PLoS ONE. 2013;8:e57257. 23468948 10.1371/journal.pone.0057257 PMC3584128

[6] Woicik PA, Urban C, Alia-Klein N, Henry A, Maloney T, Telang F, et al. A pattern of perseveration in cocaine addiction may reveal neurocognitive processes implicit in the Wisconsin Card Sorting Test. Neuropsychologia. 2011;49:1660–69. 21392517 10.1016/j.neuropsychologia.2011.02.037 PMC3100426

[7] Miyake A, Friedman NP, Emerson MJ, Witzki AH, Howerter A, Wager TD. The unity and diversity of executive functions and their contributions to complex “frontal lobe” tasks: a latent variable analysis. Cogn Psychol. 2000;41:49–100. 10945922 10.1006/cogp.1999.0734

[8] Grant DA, Berg EJ. A behavioral analysis of degree of reinforcement and ease of shifting to new responses in a Weigl-type card-sorting problem. J Exp Psychol. 1948;38:404. 18874598 10.1037/h0059831

[9] Nyhus E, Barceló F. The Wisconsin Card Sorting Test and the cognitive assessment of prefrontal executive functions: a critical update. Brain Cogn. 2009;71:437–51. 19375839 10.1016/j.bandc.2009.03.005

[10] Floresco SB, Block AE, Tse MT. Inactivation of the medial prefrontal cortex of the rat impairs strategy set-shifting, but not reversal learning, using a novel, automated procedure. Behav Brain Res. 2008;190:85–96. 18359099 10.1016/j.bbr.2008.02.008

[11] Bissonette GB, Powell EM, Roesch MR. Neural structures underlying set-shifting: roles of medial prefrontal cortex and anterior cingulate cortex. Behav Brain Res. 2013;250:91–101. 23664821 10.1016/j.bbr.2013.04.037 PMC3708542

[12] Milner B. Effects of different brain lesions on card sorting: the role of the frontal lobes. Arch Neurol. 1963;9:90–100.

[13] Mukhopadhyay P, Dutt A, Das SK, Basu A, Hazra A, Dhibar T, et al. Identification of neuroanatomical substrates of set-shifting ability: evidence from patients with focal brain lesions. Prog Brain Res. 2007;168:95–104. 10.1016/S0079-6123(07)68008-X 18166388

[14] Su C-Y, Chen H-M, Kwan A-L, Lin Y-H, Guo N-W. Neuropsychological impairment after hemorrhagic stroke in basal ganglia. Arch Clin Neuropsychol. 2007;22:465–74. 17336034 10.1016/j.acn.2007.01.025

[15] Liebermann D, Ploner CJ, Kraft A, Kopp UA, Ostendorf F. A dysexecutive syndrome of the medial thalamus. Cortex. 2013;49:40–9. 22172979 10.1016/j.cortex.2011.11.005

[16] Lie CH, Specht K, Marshall JC, Fink GR. Using fMRI to decompose the neural processes underlying the Wisconsin Card Sorting Test. Neuroimage. 2006;30:1038–49. 16414280 10.1016/j.neuroimage.2005.10.031

[17] Vatansever D, Menon DK, Stamatakis EA. Default mode contributions to automated information processing. Proc Natl Acad Sci USA. 2017;114:12821–26. 29078345 10.1073/pnas.1710521114 PMC5715758

[18] Müller VI, Langner R, Cieslik EC, Rottschy C, Eickhoff SB. Interindividual differences in cognitive flexibility: influence of gray matter volume, functional connectivity and trait impulsivity. Brain Struct Funct. 2015;220:2401–14. 24878823 10.1007/s00429-014-0797-6 PMC4981636

[19] Lee JH, Kreitzer AC, Singer AC, Schiff ND. Illuminating neural circuits: from molecules to MRI. J Neurosci. 2017;37:10817–25. 29118210 10.1523/JNEUROSCI.2569-17.2017 PMC5678014

[20] Floresco SB, Ghods-Sharifi S, Vexelman C, Magyar O. Dissociable roles for the nucleus accumbens core and shell in regulating set shifting. J Neurosci. 2006;26:2449–57. 16510723 10.1523/JNEUROSCI.4431-05.2006 PMC6793649

[21] Block AE, Dhanji H, Thompson-Tardif SF, Floresco SB. Thalamic-prefrontal cortical-ventral striatal circuitry mediates dissociable components of strategy set shifting. Cereb Cortex. 2007;17:1625–36. 16963518 10.1093/cercor/bhl073

[22] Floresco SB, Magyar O, Ghods-Sharifi S, Vexelman C, Tse MT. Multiple dopamine receptor subtypes in the medial prefrontal cortex of the rat regulate set-shifting. Neuropsychopharmacology. 2006;31:297–309. 16012531 10.1038/sj.npp.1300825

[23] Cui QL, Li Q, Geng HY, Chen L, Ip NY, Ke Y, et al. Dopamine receptors mediate strategy abandoning via modulation of a specific prelimbic cortex-nucleus accumbens pathway in mice. Proc Natl Acad Sci USA. 2018;115:E4890–99. 29735678 10.1073/pnas.1717106115 PMC6003451

[24] Roelofs TJM, Verharen JPH, van Tilborg GAF, Boekhoudt L, van der Toorn A, de Jong JW, et al. A novel approach to map induced activation of neuronal networks using chemogenetics and functional neuroimaging in rats: a proof-of-concept study on the mesocorticolimbic system. Neuroimage. 2017;156:109–18. 28502844 10.1016/j.neuroimage.2017.05.021

[25] Nakamura Y, Nakamura Y, Pelosi A, Djemai B, Debacker C, Herve D, et al. fMRI detects bilateral brain network activation following unilateral chemogenetic activation of direct striatal projection neurons. Neuroimage. 2020;220:117079. 32585345 10.1016/j.neuroimage.2020.117079

[26] Peeters LM, Hinz R, Detrez JR, Missault S, De Vos WH, Verhoye M, et al. Chemogenetic silencing of neurons in the mouse anterior cingulate area modulates neuronal activity and functional connectivity. Neuroimage. 2020;220:117088. 32592851 10.1016/j.neuroimage.2020.117088

[27] Giorgi A, Migliarini S, Galbusera A, Maddaloni G, Mereu M, Margiani G, et al. Brain-wide mapping of endogenous serotonergic transmission via chemogenetic fMRI. Cell Rep. 2017;21:910–18. 29069598 10.1016/j.celrep.2017.09.087

[28] Rocchi F, Canella C, Noei S, Gutierrez-Barragan D, Coletta L, Galbusera A, et al. Increased fMRI connectivity upon chemogenetic inhibition of the mouse prefrontal cortex. Nat Commun. 2022;13:1–15. 10.1038/s41467-022-28591-3 PMC8881459 35217677

[29] Ge F, Wang N, Cui C, Li Y, Liu Y, Ma Y, et al. Glutamatergic projections from the entorhinal cortex to dorsal dentate gyrus mediate context-induced reinstatement of heroin seeking. Neuropsychopharmacology. 2017;42:1860–70. 28106041 10.1038/npp.2017.14 PMC5520779

[30] Liu J, Wu R, Johnson B, Vu J, Bass C, Li JX. The claustrum-prefrontal cortex pathway regulates impulsive-like behavior. J Neurosci. 2019;39:10071–80. 31704786 10.1523/JNEUROSCI.1005-19.2019 PMC6978937

[31] Labouesse MA, Torres-Herraez A, Chohan MO, Villarin JM, Greenwald J, Sun X, et al. A non-canonical striatopallidal Go pathway that supports motor control. Nat Commun. 2023;14:6712. 37872145 10.1038/s41467-023-42288-1 PMC10593790

[32] Sumiyoshi A, Nonaka H, Kawashima R. Sexual differentiation of the adolescent rat brain: a longitudinal voxel-based morphometry study. Neurosci Lett. 2017;642:168–73. 28188846 10.1016/j.neulet.2016.12.023

[33] Pritschet L, Santander T, Taylor CM, Layher E, Yu S, Miller MB, et al. Functional reorganization of brain networks across the human menstrual cycle. Neuroimage. 2020;220:117091. 32621974 10.1016/j.neuroimage.2020.117091

[34] Ciampoli M, Scheggia D, Papaleo F. Automatic intra-/extra-dimensional attentional set-shifting task in adolescent mice. Front Behav Neurosci. 2021;15:704684. 34349628 10.3389/fnbeh.2021.704684 PMC8326460

[35] Lu H, Zou Q, Gu H, Raichle ME, Stein EA, Yang Y. Rat brains also have a default mode network. Proc Natl Acad Sci USA. 2012;109:3979–84. 22355129 10.1073/pnas.1200506109 PMC3309754

[36] Fredriksson I, Tsai P-J, Shekara A, Duan Y, Applebey SV, Lu H, et al. Orbitofrontal cortex and dorsal striatum functional connectivity predicts incubation of opioid craving after voluntary abstinence. Proc Natl Acad Sci USA. 2021;118:e2106624118. 34675078 10.1073/pnas.2106624118 PMC8639358

[37] Ma Z, Duan Y, Fredriksson I, Tsai P-J, Batista A, Lu H, et al. Role of dorsal striatum circuits in relapse to opioid seeking after voluntary abstinence. Neuropsychopharmacology. 2025;50:452–60. 10.1038/s41386-024-01990-4 PMC11632082 39300270

[38] Lu H, Patel S, Luo F, Li SJ, Hillard CJ, Ward BD, et al. Spatial correlations of laminar BOLD and CBV responses to rat whisker stimulation with neuronal activity localized by Fos expression. Magn Reson Med. 2004;52:1060–68. 15508149 10.1002/mrm.20265

[39] Mandeville JB, Marota JJ, Kosofsky BE, Keltner JR, Weissleder R, Rosen BR, et al. Dynamic functional imaging of relative cerebral blood volume during rat forepaw stimulation. Magn Reson Med. 1998;39:615–24. 9543424 10.1002/mrm.1910390415

[40] Duan Y, Tsai P-J, Salmeron BJ, Hu Y, Gu H, Lu H, et al. Compulsive drug-taking is associated with habenula–frontal cortex connectivity. Proc Natl Acad Sci USA. 2022;119:e2208867119. 36469769 10.1073/pnas.2208867119 PMC9897479

[41] Paxinos G, Watson C. The rat brain in stereotaxic coordinates: hard cover edition. New York: Elsevier; 2006.

[42] Roberts ME, Tchanturia K, Stahl D, Southgate L, Treasure J. A systematic review and meta-analysis of set-shifting ability in eating disorders. Psychol Med. 2007;37:1075–84. 17261218 10.1017/S0033291707009877

[43] Markicevic M, Fulcher B, Lewis C, Helmchen F, Rudin M, Zerbi V, et al. Cortical excitation:inhibition imbalance causes abnormal brain network dynamics as observed in neurodevelopmental disorders. Cereb Cortex. 2020;30:4922–37. 32313923 10.1093/cercor/bhaa084 PMC7391279

[44] Jeong M, Kim Y, Kim J, Ferrante DD, Mitra PP, Osten P, et al. Comparative three-dimensional connectome map of motor cortical projections in the mouse brain. Sci Rep. 2016;6:20072. 26830143 10.1038/srep20072 PMC4735720

[45] Uylings HB, Groenewegen HJ, Kolb B. Do rats have a prefrontal cortex? Behav Brain Res. 2003;146:3–17. 14643455 10.1016/j.bbr.2003.09.028

[46] Yuan P, Raz N. Prefrontal cortex and executive functions in healthy adults: a meta-analysis of structural neuroimaging studies. Neurosci Biobehav Rev. 2014;42:180–92. 24568942 10.1016/j.neubiorev.2014.02.005 PMC4011981

[47] Barthas F, Kwan AC. Secondary motor cortex: where ‘sensory’ meets ‘motor’ in the rodent frontal cortex. Trends Neurosci. 2017;40:181–93. 28012708 10.1016/j.tins.2016.11.006 PMC5339050

[48] Ebbesen CL, Insanally MN, Kopec CD, Murakami M, Saiki A, Erlich JC. More than just a “motor”: recent surprises from the frontal cortex. J Neurosci. 2018;38:9402–13. 30381432 10.1523/JNEUROSCI.1671-18.2018 PMC6209835

[49] Siniscalchi MJ, Phoumthipphavong V, Ali F, Lozano M, Kwan AC. Fast and slow transitions in frontal ensemble activity during flexible sensorimotor behavior. Nat Neurosci. 2016;19:1234–42. 27399844 10.1038/nn.4342 PMC5003707

[50] Wimmer RD, Schmitt LI, Davidson TJ, Nakajima M, Deisseroth K, Halassa MM. Thalamic control of sensory selection in divided attention. Nature. 2015;526:705–09. 26503050 10.1038/nature15398 PMC4626291

[51] Spellman T, Svei M, Kaminsky J, Manzano-Nieves G, Liston C. Prefrontal deep projection neurons enable cognitive flexibility via persistent feedback monitoring. Cell. 2021;184:2750–66.e17. 33861951 10.1016/j.cell.2021.03.047 PMC8684294

[52] Rodgers CC, DeWeese MR. Neural correlates of task switching in prefrontal cortex and primary auditory cortex in a novel stimulus selection task for rodents. Neuron. 2014;82:1157–70. 24908492 10.1016/j.neuron.2014.04.031

[53] Mátyás F, Komlósi G, Babiczky A, Kocsis K, Barthó P, Barsy B, et al. A highly collateralized thalamic cell type with arousal-predicting activity serves as a key hub for graded state transitions in the forebrain. Nat Neurosci. 2018;21:1551. 30349105 10.1038/s41593-018-0251-9 PMC6441588

[54] Ma Y, Hamilton C, Zhang N. Dynamic connectivity patterns in conscious and unconscious brain. Brain Connect. 2017;7:1–12. 27846731 10.1089/brain.2016.0464 PMC5312592

[55] Tsai P-J, Keeley RJ, Carmack SA, Vendruscolo JC, Lu H, Gu H, et al. Converging structural and functional evidence for a rat salience network. Biol Psychiatry. 2020;88:867–78. 32981657 10.1016/j.biopsych.2020.06.023

