# Supplementary material for: Frontostriatal regulation of brain circuits contributes to flexible decision making
Source: Neuropsychopharmacology. 2025 Feb 14;50(7):1156–66. doi: 10.1038/s41386-025-02065-8 (PMC12089345; doi:10.1038/s41386-025-02065-8)
Supplement: Supplementary file 1 — Supplementary methods and materials [file 41386_2025_2065_MOESM1_ESM.doc]

**Supplementary methods and materials**

**Frontostriatal regulation of brain circuits contributes to flexible decision making**

Ying Duan, Zilu Ma, Pei-Jung Tsai, Hanbing Lu, Xiang Xiao, Danni Wang, Aslaan Siddiqi, Elliot A Stein, Michael Michaelides, Yihong Yang

**Supplementary methods**

Stereotaxic surgery

To activate the medial prefrontal cortex (mPFC)-nucleus accumbens (NAc) pathway, we bilaterally injected adeno-associated virus (AAV) expressing the excitatory DREADD hM3Dq (AAV5-syn-hM3D-HA; Addgene, Watertown, MA) or green fluorescent protein (GFP) as control (AAV5-syn-GFP; Addgene, Watertown, MA) into the mPFC. The rats were anesthetized with isoflurane (5% induction, 2–3% maintenance; Covetrus, Portland, ME) and placed into a stereotaxic frame (Kopf, Tujunga, CA). 500 nL of AAV was injected via a 10 uL syringe (WPI, Sarasota, FL) into each side aimed at the mPFC (3.4 mm anterior to bregma, 0.7 mm from midline, 3.8 mm ventral to skull) at a rate of 50 nL/min using a micro-syringe pump (Micro4 controller; WPI, Sarasota, FL). The injection needle remained in place for 10 min before being slowly withdrawn. rats were bilaterally implanted with cannulas aimed above the NAc for later CNO or VEH administration into the NAc. MRI compatible guide cannulas (26 gauge; Plastics One, Boerne, TX) were implanted bilaterally 1 mm above the NAc (1.5 mm anterior to bregma, 2.6 mm from midline, 7 mm ventral to skull, angle 10°) and were anchored to the skull using dental cement (Parkell, Edgewood, NY). Guide cannulas were occluded by insertion of cannula blockers (Plastics One, Boerne, TX). Two weeks later (for hM3Dq or GFP expression), the rats underwent behavioral training.

Strategy acquisition and switching

Strategy acquisition and goal-directed switching behavior were assessed with a visual cue task (VCT) and a response discrimination task (RDT) using Med Associates chambers (ENV-008; Georgia, VT). Each chamber contained two levers located 7.5–8 cm above the grid floor. A food receptacle was centrally placed between two levers and food reinforcement (45 mg; TestDiet, Richmond, IN) was delivered by a pellet dispenser (ENV-203M-45; Med Associates, Georgia, VT). A light was positioned above each lever and served as a stimulus for visual-cue discrimination learning. Each chamber was illuminated by a single red house light located in the top-center of the wall opposite the levers. The behavioral training procedures including *pretraining*, *VCT* and *shift to RDT* were adapted from Floresco et al [1]*.*

*Pretraining.* On the day before initial exposure to the operant chamber, rats were given ∼ 20 reward pellets in their home cage. Under a fixed ratio 1 (FR1) schedule, rats were exposed to a pretraining sequence for 5 days, with two 90-trial sessions per day. In these sessions, only one lever was inserted, and the order of lever side was counterbalanced across sessions. Every 20 s, a trial began with illumination of the houselights. If the rat failed to respond on the lever within 10 s, the chamber darkened, and the trial was scored as an omission. If the rat responded within 10 s, a single pellet was delivered immediately and the house light remained illuminated for another 4 s. Importantly, the stimulus lights above each lever were never illuminated during these pretraining sessions. A side bias for each rat was determined on the last two days of FR1 training. If a rat made a disproportionate number of responses on one lever over the entire session (i.e., greater than a 2:1 ratio), that lever was considered its side bias; if the total number of responses on the left and right lever were comparable, the lever that a rat chose initially four or more times over seven total trials was considered its side bias.

*Visual-cue task (VCT). The* VCT session was similar to pretraining sessions except both levers were inserted into the chamber. During this phase, the rat was required to respond only on the lever with an illuminated visual-cue stimulus light. A trial began with illumination of the house light and a stimulus light above one of the levers. A correct response on that illuminated lever resulted in the delivery of one food pellet. After food delivery, the houselights remined on for 4 s, followed by return to the dark intertrial state. If the rat responded on the incorrect lever, the chamber reverted to the dark intertrial state. Failure to respond on either lever within 10 s resulted in extinguishing of the house light, and the trial recorded as an omission. In every pair of trials, the left or right stimulus light was illuminated once, and the order within the pair of trials was randomized. Trials continued until either 1) a rat had achieved criterion performance of 8 consecutive correct responses, or 2) after 200 trials. Upon completion of a training session, the rat was removed from the chamber and returned to its home cage. A second, identical session of visual-cue discrimination learning was allowed if criterion was not reached. Omission trials were not included in the trials for criterion measure.

*Response discrimination task (RDT).* This task required the animal to cease the use of a visual-cue strategy, and instead use a spatial response strategy to obtain food pellets. On the day of RDT, each rat received either CNO or VEH microinjection and returned to its home cage. After 20 min, rats were placed in the operant chamber to perform the RDT. In this task, a correct response was defined as responding on the lever opposite to its predetermined side bias, regardless of the location of the illuminated stimulus light. The lever that the animal chose, and the location of the stimulus light were recorded. Trials continued until either 1) a rat achieved criterion performance of 10 consecutive correct response, or 2) after 200 trials. Omission trials were not included in the trials for criterion measure. Only one session was allowed to assess behavioral flexibility.

*Error analysis.* We analyzed error types adapted from Floresco [1]. Errors committed during the RDT were categorized into three error subtypes. *A perseverative error* was scored when a rat responded on a lever with the stimulus light illuminated above it on trials that required the rat to press the opposite lever. For example, the rat was required to always press the left lever during the set-shift. Errors were separated into consecutive blocks of 8 trials. For example, a *perseverative error* was scored when the rat pressed the right lever when the light stimulus was on the right side. Once a rat made fewer than 6 perseverative errors in a block for the first time, all subsequent errors of this type were counted as *regressive errors*, because at this point the rat was using the original strategy less than 75% (6 out of 8) of the time. The third type of error, termed *never-reinforced errors,* was scored when a rat pressed the incorrect lever on trials where the visual-cue light was illuminated above the same lever that the rat was required to press during the set-shift. For example, a rat was required to always respond on the left lever and for half of the trials the cue was illuminated above the left lever. In this situation, a *never-reinforced* error was scored when a rat chose the right lever (i.e., a choice that was not reinforced during initial visual cue discrimination or the shift to response discrimination).

Behavioral experiment intracranial injections

Rats were habituated to the injection procedure at least three days before intracranial injections. On the test day, the injector, which was 1 mm longer than the cannula, was connected to a 10 uL Hamilton syringe (Reno, NV) via PE-50 tubing and attached to a micro-syringe pump. The injector was inserted into the cannula and injected with CNO or VEH at a rate of 500 nL/min and remained in place for additional minute to reduce diffusion up the cannula track. Cannula sites were verified by subsequent immunofluorescence staining.

fMRI experiment

*Animal preparation.* Rats were initially anesthetized with isoflurane (2.5%) in oxygen enriched air (70% N_2_ + 30% O_2_). A bolus dose of dexmedetomidine (0.015 mg/kg, intraperitoneal; Covetrus, Portland, ME) was injected 10 minutes after introduction of isoflurane. For cerebral blood volume (CBV) measurement, the tail vein was catheterized using a 23G butterfly needle filled with 30U/mL heparin (Sagent Pharmaceuticals, Plattsburgh, NY). The rats were then transferred to a customized MRI-compatible holder for head fixation. Dexmedetomidine was administered continuously (0.015 mg/kg/h) via subcutaneous infusion with an infusion pump (PHD 2000; Harvard Apparatus, South Natick, MA). Isoflurane concentration was lowered and maintained at 0.5 ~ 0.75% (0.5% step size for every 5 ~ 10 min). During imaging acquisition, which occurred at least 60 minutes after anesthesia introduction, the following parameters were monitored to ensure stable physiology: core temperature was maintained at 36.5 ± 0.5 ºC by a temperature-controlled water-heating pad; heart rate and blood oxygenation levels were continuously monitored via pulse oximetry (SA Instruments, Inc., Stony Brook, NY); and respiration rate was monitored with a MouseOx sensor (Starr Life Sciences, Oakmont, PA) beneath the animal’s chest.

*Image acquisition.* MRI data were acquired using a 9.4 Tesla scanner (Bruker, Karlsruhe, Germany) with a birdcage coil for RF excitation and single-loop surface coil for signal reception. One high-resolution T2-weighted structural image was collected using a Rapid Acquisition with Relaxation Enhancement sequence with the following parameters: repetition time (TR) = 3100 ms, echo time (TE) = 36 ms, slice thickness = 0.6 mm, slice gap = 0.1 mm, slice number = 31, field of view (FOV) = 30 × 30 cm^2^, in-plane matrix size = 256 × 256. A 10-min baseline resting-state blood oxygenation level dependent (BOLD) scan was collected using a conventional gradient echo echo-planner imaging sequence with the following parameters: TR = 1500 ms, TE = 15 ms, slice thickness = 0.6 mm, slice gap = 0.1 mm, slice number = 19, FOV = 30 × 30 cm^2^, in-plane matrix size = 80 × 80. Then, VEH or CNO were injected bilaterally into NAc and after 20 minutes, three 10-min post-injection scans were collected using the same parameters.

For neural activity measurements (CBV), a pre-contrast scan was collected using a multi-gradient echo (MGE) sequence with the following parameters: TR = 600 ms, TE = 2 ms, FOV = 30 × 30, in-plane matrix size = 128 × 128, slice thickness = 0.765 mm, slice number = 17. Feraheme (AMAG Pharmaceuticals, Waltham, WA) was injected at a dose of 15 mg/kg through a tail vein. A post-contrast MGE scan was then collected 2 minutes after Feraheme injection.

Immunofluorescence staining

To verify AAV expression and cannula placements, rats were deeply anesthetized with isoflurane (~80 s) and perfused transcardially with ∼400 mL of 0.9% saline, followed by ∼300 mL of 10% neutral formalin. For the c-Fos immunolabeling experiment, rats were deeply anesthetized with perfused 90 min after intracranial injections with CNO or VEH. Brains were removed and post-fixed in 10% formalin overnight and transferred to 30% sucrose in phosphate buffered saline (PBS) for 5 days at 4°C. Coronal sections (40 µm) were cut using a cryostat (Leica Microsystems, Deerfield, IL). Sections were washed three times in PBS with 0.1% Triton X-100 (PBS-TX), incubated for 1 h in 3% bovine serum albumin (BSA) in PBS‐TX and incubated overnight at 4°C with primary antibodies in 3% BSA in PBS‐TX. Then, the sections were rinsed in PBS-TX and incubated for 2 h in secondary antibodies diluted in 3% BSA in PBS‐TX. The dilutions of primary antibodies were as follows: anti-HA (1:100, HA-Tag (6E2) Mouse mAb #2367), anti-GFP (1:500, GFP (5G4) Mouse mAb #55494) or anti-c-Fos (c-Fos (9F6) Rabbit mAb #2250). All primary antibodies were purchased from Cell Signaling Technology (Danvers, MA). The dilutions of secondary antibodies were as follows: anti-mouse (1:500, Alexa Fluor® 488 AffiniPure Donkey Anti-Mouse IgG (H+L), 715-545-150), anti-rabbit (1:500, Alexa Fluor® 594 AffiniPure Donkey Anti-Rabbit IgG (H+L), 711-585-152). All secondary antibodies were purchased from Jackson ImmunoResearch Laboratories (West Grove, PA). Lastly, slices underwent three 10-min rinses before being mounted onto glass slides and cover-slipped with Fluoro-Gel mounting medium (Electron Microscopy Sciences, Hatfield, PA). Imaging was processed using an SLIDEVIEW VS200 Research Slide Scanner (Olympus, Tokyo, Japan).

Statistical analyses

*Behavioral data.* Repeated measures ANOVA was used for comparisons across sessions and between groups for the pretraining. Unpaired t-test was used for comparisons between groups in the VCT training. To examine the effects of mPFC-NAc activation on RDT, hM3Dq and control group were injected with either CNO or VEH 20 min before the RDT. Two-way ANOVA, with GROUP (control, hM3Dq) and TREATMENT (VEH, CNO) as between-subject factors, was used to compare number of total trials to reach criterion, percentage of total correct trials, number of incorrect trials and different type of errors. In addition, we also examined the effects of mPFC-NAc activation on VCT using a separate group of animals. Similar to the RDT test, hM3Dq and control group were injected with either CNO or VEH 20 min prior to the VCT. A two-way ANOVA, with GROUP (control, hM3Dq) and TREATMENT (VEH, CNO) as between-subject factors, was conducted to compare number of total trials required to reach the criterion, correct trials, and incorrect trials. Post-hoc comparisons were conducted using the Bonferroni test. Specifically, the comparisons between the hM3Dq-CNO and hM3Dq-VEH or hM3Dq-CNO and control-CNO groups were performed to ensure that any differences observed between the groups could be attributed to DREADD receptor activation. Additionally, the comparison between the control-CNO and control-VEH groups was used to assess whether CNO itself caused any behavioral or physiological changes, independent of DREADD activation. GraphPad Prism 10 (GraphPad Software Inc, San Diego, CA) was used for statistics and depicting graphical data. Statistical significance was preset at *p* < 0.05.

*C-Fos positive neurons analysis.* Four sections representing the NAc of each animal were selected according to a brain atlas [2] and below the cannula sites. The number of c-Fos-positive cells was semi-automatically counted using Image J software (NIH, Bethesda, MD) and was averaged for each rat. The two groups with VEH injection were combined. One-way ANOVA was used for comparing independent groups and followed by post-hoc Tukey tests. All image acquisition and analyses were conducted by investigators blinded to the experimental conditions. GraphPad Prism 10 was used for statistics and graphic results. Statistical significance was preset at *p* < 0.05.

*CBV analyses.* MGE data were preprocessed using a custom MATLAB based processing pipeline [3,4]. Specifically, voxel-wise multi-echo MRI data were fit to a single-exponential decay model:


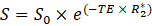


Here the
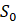
 represents MRI signal at TE = 0,
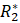
 represents the transverse-relaxation-rate. Since Feraheme is an intravascular contrast agent, changes in the
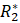
 pre- and post-MION injection (
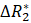
) is proportional to voxel-wise CBV [5]:


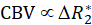


The ΔR2* was then normalized to white matter to account for inter-animal variations[3]. Voxel-wise t-tests between the VEH and CNO in hM3Dq or control group was performed separately to assess differences in CBV levels after chemogenetic activation.
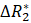
 values from clusters of significant voxels were extracted. All statistical results were corrected for multiple comparisons using Monte Carlos simulation in AFNI (corrected *p* < 0.05, uncorrected *p* < 0.05 and cluster size > 40).

*Voxel-wise analyses of functional connectivity.* Resting-state data were preprocessed using a conventional pipeline as our previous studies [4,6-8] using Analysis of Functional NeuroImages (AFNI)Co^x 9^, advanced normalization tools (ANTs, <http://stnava.github.io/ANTs/>) [10] and FMRIB Software Library (FSL, [https://fsl.fmrib.ox.ac.uk](https://fsl.fmrib.ox.ac.uk/)) [11], including distortion correction, skull stripping, motion correction, co-registration to a template, noise component removal, band-pass filtering (0.01 ~ 0.1 Hz), and spatial smoothing (FWHM = 0.8 mm). For each resting-state scan, seed-based functional connectivity maps were generated by calculating whole-brain voxel-wise Pearson’s correlation coefficients with the averaged time course of the seed; correlation values were converted to z-scores to achieve normal distribution. Functional connectivity maps were submitted to a linear mixed-effects model ANOVA, with GROUP (control, hM3Dq) as a between-subject factor, SESSION (baseline, post-injection) and TREATMENT (VEH, CNO) as within-subject factors. Post-hoc analyses were conducted in regions showing significant three-way interaction to visually demonstrate the differences in session and group. All voxel-wise statistics were corrected for whole-brain multiple comparisons (corrected *p* < 0.05, uncorrected *p* < 0.05 and cluster size > 27 voxels based on Monte Carlo simulations in AFNI). We extracted the functional connectivity values from clusters of significant brain voxels. A Principal Component Analysis (PCA) followed by K-mean clustering on functional connectivity of the above circuits was performed to determine different networks. The number of PCs was selected by Kaiser rule (eigenvalues > 1) using Prism 10. The cluster size was determined by the Elbow method [12] using R (version 4.4.1) with cluster package. Lastly, correlations between average functional connectivity in each network and set-shifting behavior (number of trials to reach criterion) were calculated. We selected this behavioral parameter as it reflected the general performance during the set-shifting procedure. Statistical significance was corrected for multiple comparisons using the Bonferroni method (p < 0.025 for functional connectivity seed with NAc and M2, p < 0.017 for functional connectivity seed with mPFC).

**Supplementary Figures**

**
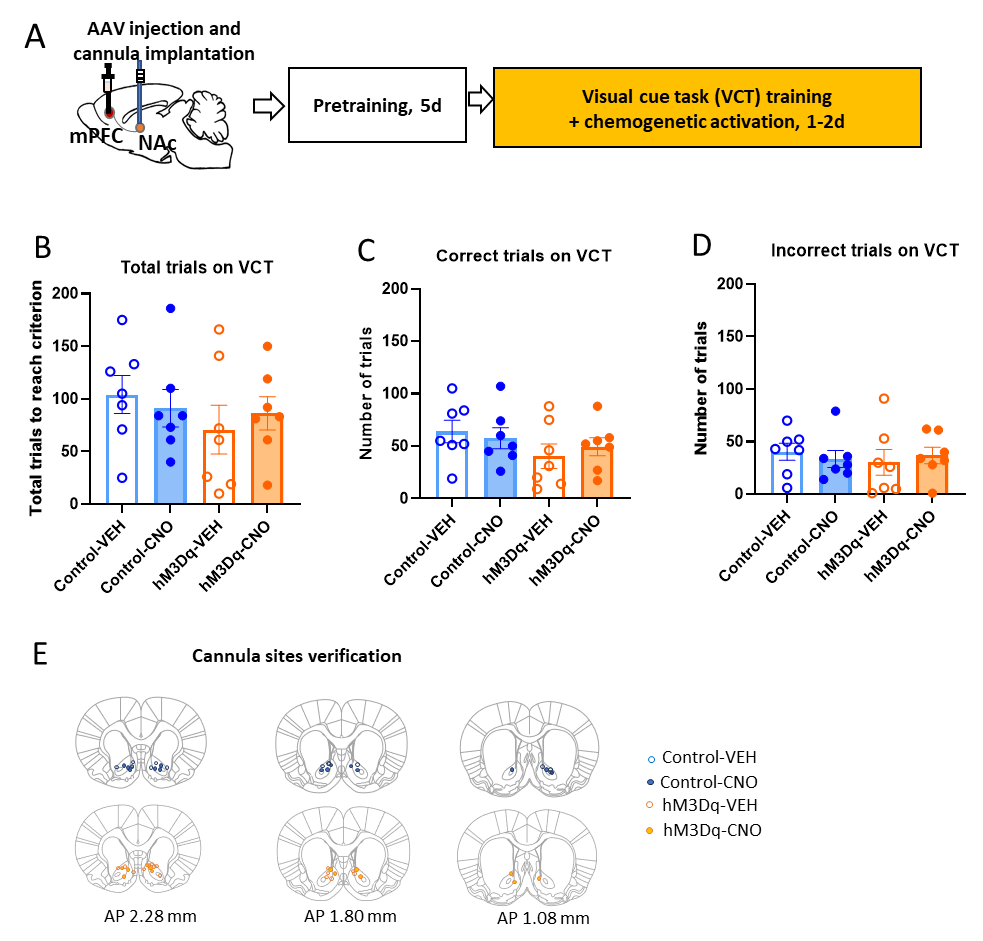
**

**Fig. S1. Effects of activation of mPFC-NAc pathway on visual cue task (VCT) learning. (A)** Experimental design and timeline. CNO or VEH was injected 20 min prior to VCT learning. **(****B)** Activation of mPFC-NAc did not affect number of total trials in VCT learning. Two-way ANOVA, group x treatment interaction: F_(1,24)_ = 0.57, p = 0.46, group effect: F_(1,24)_ = 1.00, p = 0.32; treatment effect: F_(1,24)_ = 0.0046, p = 0.95. **(C)** Activation of mPFC-NAc did not affect number of correct trials in VCT learning. Two-way ANOVA, group x treatment interaction: F_(1,24)_ = 0.55, p = 0.47, group effect: F_(1,24)_ = 2.34 , p = 0.14; treatment effect: F_(1,24)_ = 0.017, p = 0.90. **(D)** Activation of mPFC-NAc did not affect number of incorrect trials during VCT learning. Two-way ANOVA, group x treatment interaction: F_(1,24)_ = 0.51, p = 0.48, group effect: F_(1,24)_ =0.13, p = 0.72; treatment effect: F_(1,24)_ = 0.001, p = 0.99. **(E)** Cannula sites verification. N=7 per group. Error bars in the figures represent the SEM. mPFC, medial prefrontal cortex; NAc, Nucleus accumbens; VEH, vehicle; CNO, clozapine N-oxide.

**
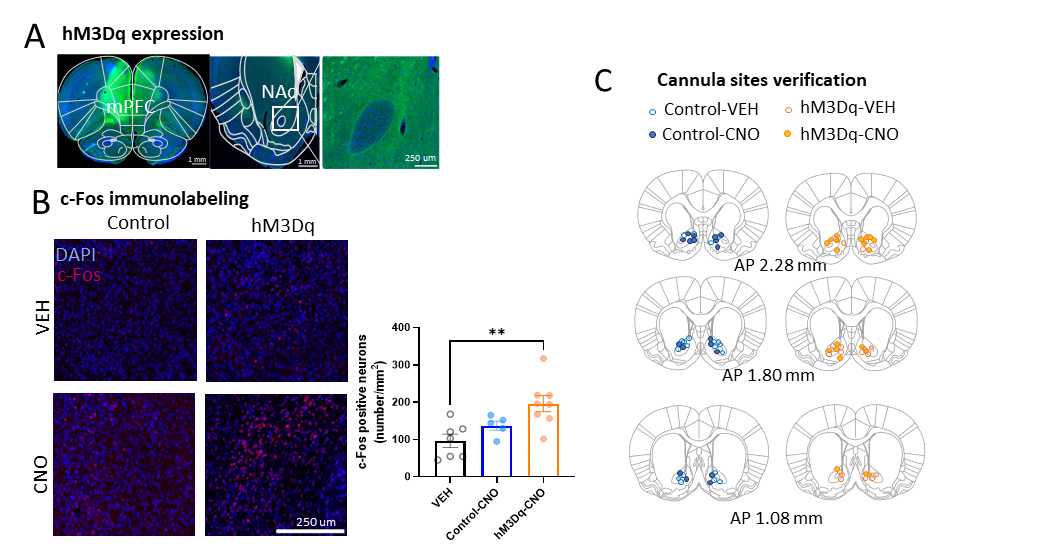
**

**Fig. S2. Verification for hM3Dq expression and cannula sites in the set-shifting test (A)** Representative images of hM3Dq expression in the mPFC and its terminals in the NAc. **(B)** Compared with the VEH group (animals expressed either excitary hM3Dq or control green fluorescent protein), CNO injection significantly increased the c-Fos in the NAc in the hM3Dq group but not the control group (n = 6-7 per group). Data were analyzed by One way ANOVA followed by Tukey tests. ** *p* < 0.01, VEH group vs. hM3Dq-CNO. **(C)** Cannula sites verification. Error bars in the figures represent the SEM. VEH, vehicle; CNO, clozapine N-oxide.

**References**

1 Floresco SB, Block AE, Tse MT. Inactivation of the medial prefrontal cortex of the rat impairs strategy set-shifting, but not reversal learning, using a novel, automated procedure. Behav Brain Res. 2008;190(1):85-96.

2 Paxinos G, Watson C. The rat brain in stereotaxic coordinates: hard cover edition*.* Elsevier; 2006.

3 Lu H, Patel S, Luo F, Li SJ, Hillard CJ, Ward BD, et al. Spatial correlations of laminar BOLD and CBV responses to rat whisker stimulation with neuronal activity localized by Fos expression. Magn Reson Med. 2004;52(5):1060-8.

4 Ma Z, Duan Y, Fredriksson I, Tsai P-J, Batista A, Lu H, et al. Role of dorsal striatum circuits in relapse to opioid seeking after voluntary abstinence. Neuropsychopharmacology. 2024:1-9.

5 Mandeville JB, Marota JJ, Kosofsky BE, Keltner JR, Weissleder R, Rosen BR, et al. Dynamic functional imaging of relative cerebral blood volume during rat forepaw stimulation. Magnetic resonance in medicine. 1998;39(4):615-24.

6 Lu H, Zou Q, Gu H, Raichle ME, Stein EA, Yang Y. Rat brains also have a default mode network. Proceedings of the National Academy of Sciences. 2012;109(10):3979-84.

7 Fredriksson I, Tsai P-J, Shekara A, Duan Y, Applebey SV, Lu H, et al. Orbitofrontal cortex and dorsal striatum functional connectivity predicts incubation of opioid craving after voluntary abstinence. Proceedings of the National Academy of Sciences. 2021;118(43):e2106624118.

8 Duan Y, Tsai P-J, Salmeron BJ, Hu Y, Gu H, Lu H, et al. Compulsive drug-taking is associated with habenula–frontal cortex connectivity. Proceedings of the National Academy of Sciences. 2022;119(50):e2208867119.

9 Cox RW. AFNI: software for analysis and visualization of functional magnetic resonance neuroimages. Computers and Biomedical research. 1996;29(3):162-73.

10 Avants BB, Tustison NJ, Song G, Cook PA, Klein A, Gee JC. A reproducible evaluation of ANTs similarity metric performance in brain image registration. Neuroimage. 2011;54(3):2033-44.

11 Smith SM, Jenkinson M, Woolrich MW, Beckmann CF, Behrens TE, Johansen-Berg H, et al. Advances in functional and structural MR image analysis and implementation as FSL. Neuroimage. 2004;23:S208-S19.

12 Nainggolan R, Perangin-angin R, Simarmata E, Tarigan AF. in *Journal of Physics: Conference Series* Vol. 1361 012015 (IOP Publishing, 2019).
